# The Korean eHealth Literacy Scale (K-eHEALS): Reliability and Validity Testing in Younger Adults Recruited Online

**DOI:** 10.2196/jmir.8759

**Published:** 2018-04-20

**Authors:** SeonYoon Chung, Bu Kyung Park, Eun-Shim Nahm

**Affiliations:** ^1^ Mennonite College of Nursing Illinois State University Normal, IL United States; ^2^ College of Nursing, Research Institute of Nursing Science Kyungpook National University Daegu Republic Of Korea; ^3^ School of Nursing University of Maryland Baltimore, MD United States

**Keywords:** eHEALS, eHealth, literacy, reliability, validity

## Abstract

**Background:**

In this digital era, eHealth literacy is an essential skill set to leverage health information available online to promote health outcomes. South Korea has an advanced health information technology infrastructure, including widespread use of the internet and mobile phones. A few studies have explored eHealth literacy in South Korea using translated versions of the eHEALS; however, they were not fully validated. A unified reliable and valid assessment tool is critical to assess and enhance the eHealth literacy level across the population.

**Objective:**

The aim was to develop a Korean version of eHealth Literacy Scale (K-eHEALS) and evaluate its reliability and validity employing healthy young adults in Korea.

**Methods:**

The K-eHEALS was developed based on eHEALS, a widely used tool that measures eHealth literacy, and was validated using a sample of 500 young adults recruited from a pool of a Korean internet survey panel. Content validity was assessed using the content validity index (CVI) for individual items and for scale. Construct validity was examined using exploratory factor analysis and hypothesis testing. The Cronbach alpha coefficient was used to determine the internal consistency and the Pearson correlation coefficient was used to evaluable the stability of the measure (n=55).

**Results:**

Both individual and scale CVIs were acceptable (individual CVIs>0.67; scale CVI=0.83). Single factors accounting for 50.3% of the variance in the scales were extracted revealing the unidimensional latent structure of K-eHEALS. Hypothesis testing showed significant association between eHealth literacy and hours of internet use per day, supporting the construct validity. Items of the K-eHEALS were internally consistent (Cronbach alpha=.88) and stable over a 1-month period (r=.754, *P*<.001).

**Conclusions:**

The findings of this study suggest that K-eHEALS is a valid and reliable measure of eHealth literacy in Korean young adults. Additional studies are needed with more diverse groups of adults in Korea.

## Introduction

In this digital era, the internet and mobile devices are integral to our daily life and the majority of the population uses the internet and social media to find health information [1]. Literacy in eHealth is an essential skillset to leverage these resources and produce better outcomes [2]. The eHealth Literacy Scale (eHEALS) is a measure to assess eHealth literacy initially developed in Canada by Norman and Skinner [3]. It is based on the Lily model that outlines six core literacies: (1) traditional literacy, (2) health literacy, (3) information literacy, (4) scientific literacy, (5) media literacy, and (6) computer literacy. The validity and reliability of eHEALS have been evaluated in numerous age groups and it has been translated globally [4-12]. However, the existing Korean versions of eHEALS (K-eHEALS) are limited in the number of items translated for use or have not been tested for their reliability and validity.

Korea has advanced health information technology infrastructure including widespread use of the internet and mobile phones [13,14]. In Korea, the concept of health literacy was introduced in the 2000s and a Korean health literacy assessment tool was first developed in 2005 [15]. This tool, Korean Health Literacy Assessment Tool, was developed by translating and modifying the Rapid Estimate of Adult Literacy in Medicine (REALM) [16] in the context of Korean culture [15]. More recently, the most frequently used tools were the Korean version of REALM, Test of Functional Health Literacy in Adults, and Newest Vital Sign [17]. These tools, however, are limited in assessing eHealth literacy.

A few studies have explored eHealth literacy in Korea. For example, Lee et al [18] examined how eHealth literacy affects communication between patients and doctors. Cho et al [19] examined the effects of cognitive factors including eHealth literacy on the health app use. More recently, eHealth literacy was assessed among nursing students in Korea [20] and relationships between eHealth literacy and health behaviors were examined in Korean adults [21]. These studies used a translated version of eHEALS [3] to measure eHealth literacy. The translated version of the eHEALS used in those studies, however, was not fully validated [18,20,21]. Not having a reliable and valid Korean version of eHealth literacy scale can significantly limit eHealth literacy in Korea that is necessary to optimize the development of interventions aimed at promoting eHealth literacy. This highlights the importance of having a unified and reliable assessment tool to compare eHealth literacy levels across the population. Thus, the purpose of this study was to develop a full eight-item K-eHEALS and evaluate its reliability and validity among healthy young adults in Korea.

## Methods

### Design

In this study, we translated eHEALS and tested the psychometric aspects of the measure using a survey. The initial data collection was conducted from September 5 to 16, 2016. To test the stability of the measure, another wave of the survey was conducted from October 3 to 10, 2016.

### Participants

The study participants were recruited (N=500) from a pool of registrants of Banana Lab, a Korean internet survey panel service agency. The survey was conducted using SurveyMonkey (SurveyMonkey Inc, Palo Alto, CA, USA). Banana Lab is an exclusive survey panel service agency working with SurveyMonkey Korea that had approximately 437,511 voluntarily registered participants in 2016. The sample size was determined based on previous studies that used a subject-to-item ratio greater than or equal to two or a sample size greater than or equal to 100 to validate a scale [22]. Potential participants who met inclusion criteria (younger adults aged 20-39 years in South Korea) received an email asking to respond to an online survey. A proportioned quota sampling by sex and age was used to minimize selection bias. For equal distribution of the younger adults, we recruited equal sample size for four sample groups by sex and age (age 20-29 male: 25%; age 20-29 female: 25%; age 30-39 male: 25%; age 30-39 female: 25%). The survey panel service agency randomly selected the potential participants from a list of registered participants with demographic information. The survey was sent out via email to potential participants in accordance to the proportion for stratified sampling, and this continued until it met our target number of participants in each quota.

Among the participants who completed the K-eHEALS, a subset of participants was randomly selected and invited to complete the same survey for test-retest reliability testing after 1 month from the initial response. If randomly selected participants did not respond, the next randomly selected participant was invited until 55 participants completed the survey. After the targeted number of 55 participants voluntarily signed and completed the survey, the online survey was closed.

### Measurement

#### Sociodemographic Attributes and Internet Use Behaviors

The survey included items on demographics and internet use behaviors, including hours of internet use per day, purpose of internet use, types of health information searched, level of trust for health information, and its usefulness. Previous studies found internet usage was related to eHealth literacy; thus, hours of internet use was measured to validate construct validity of K-eHEALS.

#### The eHEALS

The eHEALS is composed of eight items measuring eHealth literacy on a 5-point Likert scale (1=strongly disagree, 5=strongly agree), with a total score that ranges from 8 to 40, in which a higher score indicates higher literacy. The eight items measure perceived knowledge, skills, and confidence in locating, evaluating, and using electronic health information to make health decisions. The measure also includes two additional questions that are not included in the total score. These two questions assess the perception of the internet as a tool to assess health information and make decisions about health.

#### The K-eHEALS

The K-eHEALS was developed following the process of translation and adaptation of instrument proposed by the World Health Organization [23]. After we acquired permission from the original developers of the eHEALS (Dr Cameron and Dr Norman), two bilingual professionals in nursing (two of the authors) conducted forward translation independently and compared the translated instrument. Next, a bilingual expert panel, consisting of four faculty members of nursing schools in Korea and the United States and two professionals in computer and information technology, evaluated the translated instrument. All six members of the expert panel rated each item of the K-eHEALS in terms of its relevance to the underlying construct on a 4-point scale (1=not relevant, 2=somewhat relevant, 3=quite relevant, 4=highly relevant) [24]. In addition, the expert panel commented on each item if they had any suggestions or questions. Through the expert panel discussion, the translated instrument was adjusted and the complete K-eHEALS was produced. Then, back-translation of the K-eHEALS was done by an independent translator and was compared against the original eHEALS. Multimedia Appendix 1 is the final version of the K-eHEALS.

### Ethical Considerations

The study was reviewed and approved as an exempt study by the corresponding author’s Institutional Review Boards (1041078-201608-HRSB-151-01). The data collection was conducted by a third party, Banana Lab, a Korean internet survey panel service agency; the researchers were not in direct contact with the potential participants and received deidentified survey data. Eligible individuals who were interested in the study received an email including a link to the online consent form, which explained the study and other information listed in the face-to-face consent form. On review of the form, those who agreed to participate in the survey typed their name and clicked on the “I agree” button to consent to participate in the study and proceed to the survey.

### Data Analysis

#### Validity

##### Content Validity

To determine the content validity index (CVI) for individual items, six members of the expert panel rated each item in terms of its relevance to the underlying construct on a 4-point scale. Then, individual CVI was computed for each item as the number of experts giving a rating of either 3 or 4, divided by the number of experts (the proportion of agreement about relevance). The CVI for the scale was calculated as the mean of the individual CVIs for all items on the scale [25]. An individual CVI higher than 0.78 was considered excellent; a scale CVI higher than 0.80 was considered acceptable [26].

##### Construct Validity: Exploratory Factor Analysis

After content validity was confirmed, the Korean eHEALS was administered to young adults through an online survey. Exploratory factor analysis was conducted to ensure the construct validity [27]. Sufficiency of the sample size relative to the number of items was determined using the Kaiser-Meyer-Olkin value (>0.70) and factorability of the data were evaluated based on the Bartlett test of sphericity. A scree plot and the eigenvalue (>1) were used to determine the number of factors to be extracted [28].

##### Construct Validity: Hypothesis Testing

Construct validity was also assessed using a hypothesis-testing approach [27]. Based on prior studies [2,12,29], we hypothesized that young adults who used the internet for more hours would have higher eHealth literacy scores. Both analysis of variance and Tukey post hoc analyses were used to test the association between duration of internet use and eHealth literacy.

#### Reliability

##### Interitem Consistency

Interitem reliability was calculated by Cronbach alpha. A value of .70 or higher was considered acceptable [30].

##### Stability of the Measure

Test-retest reliability testing was conducting using Pearson correlation.

## Results

### Characteristics of the Young Adult Participants

The total number of young adults included in the study was 500. Half of the participants were male (n=250). Half of the participants were in their twenties, whereas the other half were in their thirties. More than two-thirds of the participants were single (72.6%, 363/500) and had at least some university education (437/500, 87.4%) (Table 1). The highest percentage of participants used the internet for 1 to 3 hours per day (personal computer; PC: 38.6%, 193/500; portable device: 49.2%, 246/500), followed by 4 to 7 hours per day (PC: 29.6%, 148/500; portable device: 23.6%, 118/500). More than half of the participants (54.6%, 273/500) used the internet to search for information. Types of health information searched for included healthy lifestyle (45.0%, 225/500), disease (32.8%, 164/500), and treatment and medicine (15.8%, 79/500). More than half (53.6%, 268/500) of the participants neither trusted nor distrusted health information online, but 64.8% (324/500) reported the internet is useful in making decisions about health and 64.4% (322/500) reported the internet is important to have access to health resources. The mean total score on the K-eHEALS was 28.06 (SD 4.80, range 8-40) (Table 1). The mean of items in the K-eHEALS was 3.51 ranging from 3.31 to 3.68 (item range 1-5) (Table 2).

### Content Validity

The individual CVI were excellent, scoring higher than 0.78 for all items except for item 3 (“I know how to find helpful health resources on the internet”) and item 6 (“I have the skills I need to evaluate the health resources I find on the internet”). Although scoring was lower than 0.78 for two items, the experts commented that the underlying construct for these two items were apparent but they gave them a low individual CVI score because of the low fluency of the translation. Therefore, the K-eHEALS was used after editing items 3 and 6 per the experts’ suggestions. The scale CVI was acceptable, scoring higher than 0.80 (scale CVI=0.83) (Table 3).

**Table 1:** Characteristics of the young adult participants (N=500).

Characteristics		n (%)
**Gender**		
	Male	250 (50.0)
	Female	250 (50.0)
**Age group (years)**		
	20-29	250 (50.0)
	30-39	250 (50.0)
**Education level**		
	High school diploma	166 (33.2)
	University degree and above	334 (66.8)
**Marital status**		
	Single	363 (72.6)
	Married	137 (27.4)
**Internet use on personal computer per day (hours)**		
	<1	55 (11.0)
	1-3	193 (38.6)
	4-7	148 (29.6)
	>8	104 (20.8)
**Internet use on phone per day (hours)**		
	l<1	77 (15.4)
	1-3	246 (49.2)
	4-7	118 (23.6)
	>8	59 (11.8)
**Purpose of internet use**		
	Social networking service (eg, Kakaotalk,^a^ Instagram)	171 (34.2)
	Searching information	273 (54.6)
	Game	30 (6.0)
	Others	26 (5.2)
**Health information searched for**		
	Disease	164 (32.8)
	Healthy lifestyle	225 (45.0)
	Medicine	36 (7.2)
	Treatment	43 (8.6)
	Medical personnel	4 (0.8)
	Others	28 (5.6)
**Engine used to search health information**		
	Google	38 (7.6)
	Naver	423 (84.6)
	Daum	32 (6.4)
	YouTube	5 (1.0)
	Others	2 (0.4)
**Level of trust for health information**		
	Strongly trust	11 (2.2)
	Quite trust	187 (37.4)
	Neutral	268 (53.6)
	Quite distrust	31 (6.2)
	Never trust	3 (0.6)
**How useful do you feel the internet is in helping you in making decisions about your health?**		
	Not useful at all	4 (0.8)
	Not useful	29 (5.8)
	Unsure	120 (24.0)
	Useful	324 (64.8)
	Very useful	23 (4.6)
**How important is it for you to be able to access health resources on the internet?**		
	Not important at all	4 (0.8)
	Not important	22 (4.4)
	Unsure	105 (21.0)
	Important	322 (64.4)
	Very important	47 (9.4)

^a^Kakaotalk is one of the most popular messenger apps in South Korea.

**Table 2:** Total and item means for the K-eHEALS in young adult participants (N=500).

K-eHEALS items	Mean (SD)
1. I know what health resources are available on the internet	3.53 (0.76)
2. I know where to find helpful health resources on the internet	3.47 (0.80)
3. I know how to find helpful health resources on the internet	3.59 (0.80)
4. I know how to use the internet to answer my questions about health	3.68 (0.77)
5. I know how to use the health information I find on the internet to help me	3.62 (0.77)
6. I have the skills I need to evaluate the health resources I find on the internet	3.31 (0.85)
7. I can tell high quality from low quality health resources on the internet	3.41 (0.87)
8. I feel confident in using information from the internet to make health decisions	3.44 (0.81)
Total means	3.51

### Construct Validity-Exploratory Factor Analysis

**Figure 1:**
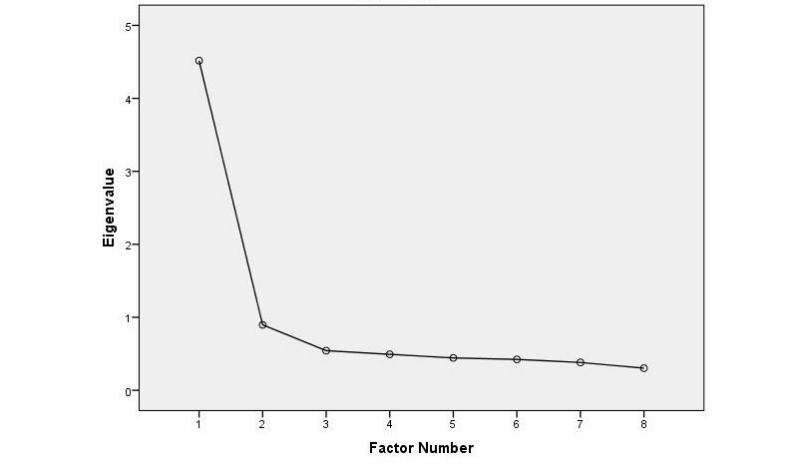
Scree Plot of the K-eHEALS.

The results supported the validity of the K-eHEALS. The Bartlett test of sphericity was significant (χ^2^_28_=1859.0, *P*<.001) suggesting the factorability of the correlation matrix. The results of the Kaiser-Meyer-Olkin test (0.91) was high, showing adequate sampling relative to the number of items present. Based on the initial eigenvalue (4.52) and the scree plot that was suggestive of a unidimensional latent structure (Figure 1), a single factor was retained. In this single factor model, the sum of squared loadings of the eight items on the extracted factor based on maximum likelihood method was 4.02, explaining 50.3% of the variance in the scale (Table 4).

### Construct Validity-Hypothesis Testing

The results from hypothesis testing further supported the construct validity of the K-eHEALS (Table 5). There was a significant association between eHealth literacy and the hours of internet use per day using PC (*F*_4,106.3_=5.608, *P*<.001). Post hoc analysis showed that the difference was evident between adults using internet on PC for less than 1 hour per day compared to other groups that used more than 1 hour: 1 to 3 hours, 4 to 7 hours, or 8 to 11 hours. Similarly, there was significant association between eHealth literacy and hours of internet use per day using a portable device (*F*_4,98.0_=4.610, *P=*.002). Post hoc analysis showed that the difference in mean eHealth literacy was evident between adults using internet on a portable device for less than 1 hour per day compared to other groups that used more than 1 hour: 1 to 3 hours, 4 to 7 hours, or more than 12 hours. The difference was also shown between those using the Web for 8 to 11 hours and more than 12 hours.

**Table 3:** Individual content validity index (CVI) and scale CVI scores for the Korean eHEALS (K-eHEALS).

K-eHEALS Items	Expert 1	Expert 2	Expert 3	Expert 4	Expert 5	Expert 6	Individual CVI
1. I know what health resources are available on the internet	3	3	3	3	2	4	0.83
2. I know where to find helpful health resources on the internet	3	4	3	3	2	4	0.83
3. I know how to find helpful health resources on the internet	4	4	2	4	2	4	0.67
4. I know how to use the internet to answer my questions about health	4	4	4	3	2	4	0.83
5. I know how to use the health information I find on the internet to help me	3	4	4	3	4	4	1.00
6. I have the skills I need to evaluate the health resources I find on the internet	2	4	2	3	3	3	0.67
7. I can tell high quality from low quality health resources on the internet	3	4	2	4	4	4	0.83
8. I feel confident in using information from the internet to make health decisions	4	4	3	4	3	4	1.00
Scale CVI							0.83

**Table 4:** Factor loadings and factor score coefficients for the K-eHEALS and single extracted factor (N=500).

K-eHEALS Items	Factor loadings^a^
1. I know what health resources are available on the internet	0.758
2. I know where to find helpful health resources on the internet	0.737
3. I know how to find helpful health resources on the internet	0.762
4. I know how to use the internet to answer my questions about health	0.787
5. I know how to use the health information I find on the internet to help me	0.705
6. I have the skills I need to evaluate the health resources I find on the internet	0.623
7. I can tell high quality from low quality health resources on the internet	0.616
8. I feel confident in using information from the internet to make health decisions	0.664
Sums of squared loadings	4.022

^a^ Extraction method: maximum likelihood. One factor extracted, four iterations required.

**Table 5:** Participant eHealth literacy by hours of internet use (N=500).

Hours of internet use		N	Mean (SD)	*P* value	*F* (*df1*, *df2*)	*P* value
**Internet use on personal computer (hours)**		500	28.06 (4.81)		5.608 (4,106.30)	<.001
	<1	55	25.60 (5.91)	—		
	1-3	193	27.68 (5.30)	.032^a^		
	4-7	148	28.63 (3.89)	.001^a^		
	8-11	83	29.34 (3.41)	<.001^a^		
	>12 hours	21	28.90 (5.09)	.050^a^		
**Internet use on phone (hours)**		500	28.06 (4.81)		4.610 (4,98.03)	.002
	<1	77	26.30 (5.90)	—		
	1-3	246	28.13 (4.07)	.026^b^		
	4-7	118	28.86 (4.43)	.002^b^		
	8-11	32	26.81 (6.90)	.986^b^		
	>12	27	30.48 (4.52)	.001^b^		

^a^Versus <1 hour internet use on personal computer.

^b^Versus <1 hour internet use on phone.

### Reliability

Regarding interitem reliability, the calculated Cronbach alpha coefficient was .88, suggesting that the K-eHEALS was internally consistent. The measure also showed stability over time as evidenced by high test-retest reliability (*r*=.754, *P*<.001).

## Discussion

### Principal Findings

This study is the first attempt to translate a full version of eHEALS into Korean and to test its psychometric aspects. Although additional psychometric testing is necessary to further establish validity of this measure in this population, results of this study are promising and support the validity and reliability of K-eHEALS. Content validity was acceptable (individual CVIs>0.67, scale CVI=0.83), and so was construct validity as supported by unidimensional latent structure of K-eHEALS and significant association between eHealth literacy and hours of internet use per day. In terms of reliability, the items of K-eHEALS were internally consistent (Cronbach alpha=.88) and stable over a 1-month period (*r*=.754, *P*<.001). Therefore, the results of this study reveal that the translated K-eHEALS is reliable.

The eHEALS is a measure that has been globally validated in multiple languages, including Japanese [10], Dutch [5], Spanish [11], Chinese [9], German [6], Italian [4], Iranian [8], Hebrew [12], and Turkish [7]. In Korea, several studies have used a translated version of eHEALS, but those studies did not provide psychometric evaluation of the eHEALS in Korean [18,20,21].

Content validity of K-eHEALS reported here was evaluated by six experts and it showed good scale CVI and individual CVI except for two items. However, previous studies on various language versions of eHEALS did not conduct content validity or report the CVI score (all six). Only one study, an Iranian version of eHEALS [8], measured face validity by four experts, but did not evaluate the CVI score. Evaluation of content validity is proposed for future psychometric studies to enhance construct validity of all instruments [26,31].

Construct validity of the K-eHEALS was evaluated by exploratory factor analysis and hypothesis testing. The K-eHEALS showed a monofactorial unidimensional structure and explained 50.3% of the variance in the measure. This finding supports previous studies that yielded a single factor solution explaining from 52.6% (Spanish version) to 70.5% (Iranian version) of variance in the measure.

Internal consistency of the K-eHEALS was .88, which was comparable with previous findings ranging from .78 (Turkish version) to .93 (Japanese version). Test-retest reliability of the K-eHEALS showed stability (*r*=.754, *P*<.001), with the *r* coefficient within range of previous studies (*r*=.63 for Japanese version and *r*=.96 for Iranian version). Therefore, the K-eHEALS is a reliable and valid tool compared to other translated versions of eHEALS.

In South Korea, a higher percentage of Koreans use the internet to search and read health information (66.4%) compared to those who seek information from mass media (40.8%) or a health care provider (11.8%) [32]. Moreover, there is an increasing interest in the use of personal health records (PHRs). For young adults, eHealth literacy is essential for effective use of online resources and their PHR to assist in self-management and promote health conditions. Acknowledging the importance of eHealth literacy, the US Office of Disease Prevention and Health Promotion specified the need for increased heath literacy, access to the internet, and use of health information technologies to promote health of the public in *Healthy People 2020*, a 10-year national objective for improving public health in the United States [33]. The Health Information Technology for Economic and Clinical Health (HITECH) Act [34] also encourages health care institutions to use electronic health records (EHRs), PHRs tethered to EHRs, and related technology for meaningful use of EHRs to improve health [35]. However, there is no prominent national initiative available to promote eHealth literacy in South Korea. More research aimed at understanding of the current level of eHealth literacy in this population is needed to develop more effective eHealth interventions to promote the health of the public. The K-eHEALS, which is a reliable and valid measure, can significantly contribute to these efforts [18].

### Limitations

A main limitation of our study is that the participants were recruited from a pool of registrants of an internet survey panel service agency who are likely active online users and this group sample may not be representative of the general young adult population. Moreover, the results cannot be generalized to older adults because this was studied in young adults. Further studies employing diverse age groups are needed to address this issue.

### Conclusion

To promote eHealth literacy, researchers and health care providers should first understand the eHealth literacy of the individuals. The psychometric findings from this study suggest that K-eHEALS is a reliable and valid measure of eHealth literacy in young Korean adults who are active online users. We hope K-eHEALS can help Korean researchers who conduct studies in eHealth by providing a reliable and valid measure that can properly gauge participants’ eHealth literacy and develop optimal interventions.

## Appendix

Multimedia Appendix 1 Korean version of eHEALS (K-eHEALS).

## Abbreviations

CVI: content validity index
eHEALS: eHealth Literacy Scale
EHR: electronic health record
K-eHEALS: Korean version of eHealth Literacy Scale
PHR: personal health record
REALM: Rapid Estimate of Adult Literacy in Medicine
