# Supplementary material for: The Korean eHealth Literacy Scale (K-eHEALS): Reliability and Validity Testing in Younger Adults Recruited Online
Source: J Med Internet Res. 2018 Apr 20;20(4):e138. doi: 10.2196/jmir.8759 (PMC5935806; doi:10.2196/jmir.8759)
Supplement: Multimedia Appendix 1 [file jmir_v20i4e138_app1.pdf]

인터넷 건강정보 활용능력 (K-eHEALS)

1. 건강과 관련된 결정을 내릴 때 인터넷이 얼마나 유용하다고 생각합니까?
① 전혀 유용하지 않다.
② 유용하지 않다.
③ 잘 모르겠다.
④ 유용하다.
⑤ 매우 유용하다.

2. 건강관련 정보를 얻기 위해 인터넷을 이용할 수 있다는 것이 얼마나 중요합니까?
① 전혀 중요하지 않다.
② 중요하지 않다.
③ 잘 모르겠다.
④ 중요하다.
⑤ 매우 중요하다.

3. 나는 인터넷의 **어떤** 건강정보를 이용할 수 있는지 알고 있다.
① 매우 동의하지 않는다
② 동의하지 않는다.
③ 잘 모르겠다.
④ 동의한다.
⑤ 매우 동의한다.

4. 나는 인터넷의 유용한 건강정보를 **어디서** 찾아야 하는지 알고 있다.
① 매우 동의하지 않는다
② 동의하지 않는다.
③ 잘 모르겠다.
④ 동의한다.
⑤ 매우 동의한다.

5. 나는 인터넷의 유용한 건강정보를 **어떻게** 찾아야 하는지 알고 있다.
① 매우 동의하지 않는다
② 동의하지 않는다.
③ 잘 모르겠다.
④ 동의한다.
⑤ 매우 동의한다.

6. 나는 건강과 관련된 궁금증을 해결하기 위해 인터넷을 **어떻게 이용**해야 하는지 알고 있다.
① 매우 동의하지 않는다
② 동의하지 않는다.
③ 잘 모르겠다.
④ 동의한다.
⑤ 매우 동의한다.

7. 나는 인터넷에서 찾은 **건강정보**를 어떻게 이용해야 나에게 도움이 되는지 알고 있다.
① 매우 동의하지 않는다
② 동의하지 않는다.
③ 잘 모르겠다.
④ 동의한다.
⑤ 매우 동의한다.

8. 나는 인터넷에서 찾은 건강관련 정보를 **평가**할 수 있다.
① 매우 동의하지 않는다
② 동의하지 않는다.
③ 잘 모르겠다.
④ 동의한다.
⑤ 매우 동의한다.

9. 나는 인터넷에서 찾은 건강관련 정보의 **질이 높고 낮음**을 구별할 수 있다.
① 매우 동의하지 않는다
② 동의하지 않는다.
③ 잘 모르겠다.
④ 동의한다.
⑤ 매우 동의한다.

10. 나는 건강과 관련된 결정을 내리기 위해 인터넷에서 찾은 정보를 이용하는데 **자신이 있다**.
① 매우 동의하지 않는다
② 동의하지 않는다.
③ 잘 모르겠다.
④ 동의한다.
⑤ 매우 동의한다.
